# Before and after the ban: energy drink consumption among physically active Polish youth

**DOI:** 10.5114/biolsport.2025.156871

**Published:** 2025-09-09

**Authors:** Dominika Granda, Jadwiga Malczewska-Lenczowska, Olga Surała, Beata Szczepańska

**Affiliations:** 1 Department of Nutrition Physiology, Institute of Sport—National Research Institute, Warsaw, Poland

**Keywords:** Energy drinks, Adolescents, Ban, Policy, Public health, Athletes

## Abstract

On January 1, 2024, a nationwide ban on sales of energy drinks (EDs) to individuals under 18 came into effect in Poland. The aim of this study was to compare ED consumption, motives, and contexts of use before and after implementation of the sales ban. Two studies with similar methodology were conducted in independent samples: the first in 2022 (n = 1530, adolescents participating in extracurricular sports activities, aged 10–14 years) and the second in 2025 (n = 1083, adolescents from Handball Training Centres, aged 11–15 years). Both studies were anonymous, included participants from all provinces and applied the computer-assisted web interview method. The prevalence of ED consumption among adolescents decreased significantly from 46.4% in 2022 to 19.1% in 2025 following implementation of the sales ban for minors (p < 0.001; OR = 0.27, 95%CI: 0.23–0.33). Before and after the ban, the percentage of ED consumers increased with age. Taste and consuming EDs with friends were the most frequently cited motives and circumstances in both studies. The significant decline in the proportion of ED consumers suggests that the implemented policy may have been effective, albeit to a limited extent. The ban did not alter the circumstances and motives for consumption. To our knowledge, this is the first study to directly compare ED consumption before and after implementation of such a policy among physically active adolescents. Research on a representative youth sample, including adolescents who do not train, is needed to assess the scale of the problem and the effectiveness of the sales ban.

## INTRODUCTION

Adolescence is a period of intense physical, psychological, and emotional development. During this time, the nervous system is still maturing and remains particularly vulnerable to various lifestyle-related external factors [1]. While balanced nutrition and regular physical activity can have a beneficial impact, other factors such as caffeine or alcohol may exert adverse effects [2]. The teenage years are also a time marked by experimentation and heightened susceptibility to peer pressure. One increasingly common and potentially harmful behaviour among adolescents is the consumption of energy drinks (EDs), which typically contain ≥ 80 mg of caffeine per package. According to the 2013 report by the European Food Safety Authority (EFSA), children and adolescents were the primary consumers of EDs and consumed them more frequently than adults [3]. This trend raises significant public health concerns, as excessive intake of EDs has been associated with a range of negative outcomes, including mental health problems (e.g., anxiety, sleep disturbances), an increased likelihood of engaging in risky behaviors (such as substance use or aggressive conduct), and negative impacts on cardiovascular health and academic performance [4–8].

Physically active children and adolescents appear to be particularly vulnerable to high ED consumption. This may be attributed to several factors. First, research has shown that caffeine can enhance acute aerobic exercise performance [9], and it is listed in Group A of the Australian Institute of Sport’s (AIS) supplement classification system, indicating strong scientific support for its use in certain athletic contexts. However, the position stand of the International Society of Sports Nutrition (ISSN) clearly states that young athletes under 12 years of age should not consume EDs at all, and that adolescents aged 12–18 should only consider using EDs around training under direct parental supervision [10]. Another contributing factor may be a lack of knowledge. Studies have indicated that many physically active adolescents are unable to distinguish between isotonic drinks—recommended for prolonged physical activity—and EDs [11], and young people are often unaware of the potential adverse effects of excessive consumption of EDs [12]. Considering these factors, along with the fact that no safe caffeine intake level has been established for children and adolescents, research focused on physically active youth is both necessary and timely.

This issue has been recognized by several countries, which have implemented various strategies to curb ED consumption among children and adolescents. These measures include taxation, school-based bans, mandatory labelling requirements, restrictions on marketing and advertising, and, in some cases, legal prohibitions on the sale of EDs to minors [13]. In Poland, a nationwide ban on the sale of EDs to individuals under the age of 18 came into effect on January 1, 2024 [14]. Since then, three studies on EDs consumption in Poland have been published, two in a group of students [15, 16], and the other among adults [17]. All these studies included untrained individuals. However, to date, no studies have been conducted to assess the effectiveness of this regulatory measure. The aim of this study was to conduct a comparative analysis of ED consumption among adolescents in Poland participating in organized sports activities, before and after the implementation of the sales ban on EDs to minors.

## MATERIALS AND METHODS

Two studies using a similar methodology were conducted: first in 2022 [11], and the second in 2025. It is important to note that the participants in the studies conducted before and after the implementation of the sales ban were not the same individuals. In the first study, conducted in 2022, data were collected from participants of the School Sports Club (Polish: Szkolny Klub Sportowy, SKS), a programme of systematic sports and recreation activities addressed to all Polish children and youth. Given that access to the SKS cohort was no longer possible because the program was discontinued in 2024, and in order to incorporate data from the most comparable population (namely, youth engaged in organized, extracurricular sports activities offered nationwide), we recruited children registered in the Handball Training Centres (HTC) programme. The HTC initiative is a structured, nationwide handball training programme designed for children and adolescents from grades 4 to 8 of primary school, which covers 5300 students from all across Poland. It was launched in September 2015 by the Polish Minister of Sport and Tourism. The following study was approved by the Ethics Committee of the Institute of Sport—National Research Institute, Poland (KEBN-23-84-TMK), and was carried out in accordance with the Declaration of Helsinki (2000) of the World Medical Association.

### Study design

**FIG. 1:**
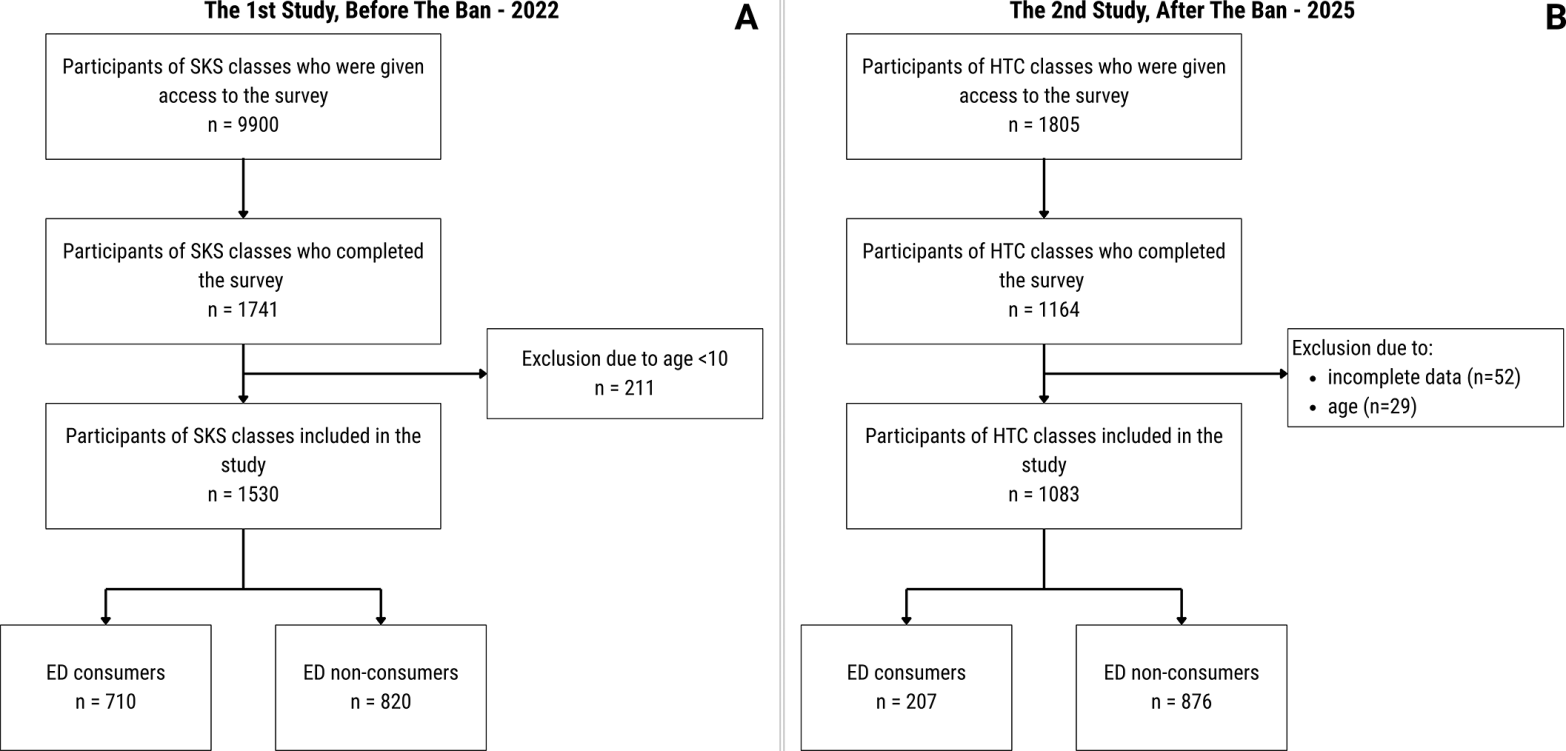
Flow chart of the participants recruitment and enrolment in the two studies – before (A) and after (B) the implementation of the energy drink (ED) sales ban to minors. Abbreviations: SKS – School Sports Club, HTC – Handball Training Centres

The methodological details of the 2022 study have been reported elsewhere [11]. Briefly, a nationwide web-based survey was conducted among primary school students aged 10–14 years participating in an extracurricular school sports programme. Data were collected between October and November 2022, i.e., prior to the national ban on the sale of EDs to minors. The questionnaire was completed anonymously during additional sports classes, with teachers facilitating access to the online survey. Approximately 9900 students were reached and 1741 provided complete responses. Regarding the second study: a nationwide online survey was carried out between May and August 2025, one and a half year after the restriction on selling EDs to minors was implemented. The questionnaire was addressed to physically active children aged 11 to 15 years, corresponding to the SKS cohort examined in the initial study from 2022. Therefore, the survey conducted in 2025 focused on primary school students in grades 4 to 8 who participated in the HTC program. The questionnaire together with information for study participants was made available to 1805 students. Data were collected during extracurricular sport classes and in summer sport camps. Parents provided consent, and students gave assent, within the standard consent process for class participation. Upon our request, the teachers conducting the extracurricular handball classes provided students with a QR code that linked to the survey. The students completed the survey anonymously on their mobile phones, but in the presence of a teacher who could answer any questions in case of doubt. Access to the QR codes was restricted to teachers employed within the program, which minimized the possibility of children outside the target group completing the survey. In total 1164 has responded to our request (Figure 1). Therefore, the response rate was 64.5%. We had to exclude 52 participants due to incomplete data and 29 due to age < 11 or > 15 years. After all exclusions, the data of 1083 students were included in the final study sample. The number of participants by province is shown in Table S1 in the Supplementary Materials.

### Questionnaire for Data Collection on Energy Drink Use

The questionnaire employed in the 2025 study was largely based on the instrument used in 2022, which has been described in greater detail elsewhere [11]. This decision was made deliberately in order to enable a direct comparison of the results from both studies. Additionally, our questionnaire incorporated elements of the validated EFSA questionnaire from 2013 [3]. In brief, the questionnaire included both single- and multiple-choice closed-ended questions designed to assess adolescents’ habits related to the consumption of EDs. Questions concerning ED consumption were preceded by demographic items, such as year of birth, sex, voivodeship (province), number of siblings, and place of residence (urban/rural). Respondents’ knowledge about EDs (EDs recognition) was assessed with the question: “In your opinion, are energy drinks and isotonic drinks the same beverages?” with three possible response options: “Yes,” “No,” and “I do not know/It is difficult to say.” After this question, a short definition of EDs was presented before proceeding with the rest of the questionnaire. The survey also included a question on the frequency of ED consumption during the past six months. Students were asked to select one of the following response options: “less than once a month”, “1–3 times a month”, “1–2 times a week”, “3–4 times a week”, “5–6 times a week”, “once a day or more often”. Based on this question, respondents were classified as ED consumers or non-consumers. Individuals who selected the option “less than once a month” were treated as non-consumers. This approach differs from that used in the 2022 study, where classification was based on the question “Have you consumed an energy drink in the past three days?”, with affirmative responses categorizing respondents as consumers. In the present 2025 study, the definition was deliberately modified following the results of a pilot study conducted among a smaller group (n = 20) of physically active adolescents. In that pilot, the three-day reference period did not identify any consumers, which would have prevented analysis of the motives and contexts of ED use, one of the key aims of the study. Anticipating a lower prevalence of consumption, we therefore adopted a broader definition of ED consumer status to ensure that the study could capture relevant patterns and determinants of use while maintaining methodological rigor. The English-language version of the questionnaire used in the 2025 study has been included in the supplementary materials.

### Statistical analysis

Qualitative data were presented as frequency and percentage distributions, while quantitative data were reported as means with standard deviations. For quantitative variables, the normality of distribution was assessed using the Shapiro-Wilk test. If the distribution was normal, the means between the two groups were compared using the independent Student’s t test. If the distribution deviated from normality, the Mann-Whitney U test was used to compare the two groups. Pearson’s chi-square test (χ^2^) was used to examine categorical study variables. In cases where subgroup counts were too small (< 5 per cell), the χ^2^ test was replaced with Fisher’s exact test to ensure the validity of the analysis. To identify the significant pairs between groups, multiple column comparisons using RxC with Benjamini-Hochberg correction were applied. Unadjusted odds ratios (ORs) with 95% confidence intervals were calculated from 2 × 2 contingency tables using the Wald method; to quantify the risk of being a consumer in 2022 relative to 2025, we computed the risk (prevalence) ratio from the corresponding 2 × 2 table with log-transformed Wald confidence intervals. A p-value < 0.05 was considered statistically significant. Statistical analysis were performed using Statistica (Verion 13) software.

## RESULTS

### The After the Ban study – characteristics of the study group and subgroup comparisons

In the study conducted in 2025, the distribution of girls and boys was nearly equal (50.6% vs 48.3%). Twelve students did not specify their sex and were therefore excluded from further analyses (Table 1). The most common age groups were 13- and 14-year-olds, followed by 11- and 12-year-olds. The smallest group was that of 15 years old. Most respondents lived in urban areas (75.1%) and had siblings (84.1%), while 15.9% were the only child. In the entire group (n = 1083), most students distinguished EDs from isotonic drinks (77.6%), 19.9% chose the response “difficult to say”, and 2.6% indicated that they considered them to be the same. Overall, 19.1% of participants were consumers of EDs. The descriptive characteristics of the students surveyed in 2022 have been published elsewhere [11].

**TABLE 1:** Descriptive characteristics of total sample studied in 2025, after implementation of the ban on sales to minors (n = 1083)

	N (%)
**Sex**	
Girls	548 (50.6)
Boys	523 (48.3)
Other/Prefer not to answer*	12 (1.1)

**Age (years of age)**	
11	177 (16.3)
12	166 (15.3)
13	298 (27.5)
14	344 (31.8)
15	98 (9.0)

**Residency**	
Urban	813 (75.1)
Rural	270 (24.9)

**Siblings**	
None	172 (15.9)
Older siblings	409 (37.8)
Younger siblings	383 (35.4)
Both older and younger siblings	119 (11.0)

Energy drink recognition**	
Yes	840 (77.6)
No	28 (2.6)
Do not know/Unsure	215 (19.9)

**Energy drink consumer**	
Yes	207 (19.1)
No	876 (80.9)

* Participants not included in further statistical analysis;

** Response to the following question “In your opinion, are energy drinks and isotonic drinks the same beverages?”.

In the 2025 study, consumers of EDs were significantly older than non-consumers (13.6 ± 1.0 vs 12.9 ± 1.2 years, p < 0.001; Table 2). No statistically significant differences were found in terms of sex and place of residence. Consumers were significantly more likely than non-consumers to confuse EDs with isotonic drinks (5.9% vs 1.7%). The EDs consumer group included significantly more only children (21.3% vs 14.6%) and fewer students with younger siblings (28.8% vs 36.9%) than the non-consumers group.

**TABLE 2:** Descriptive comparison of energy drink (ED) consumers and non-consumers from the study after the implementation of the ban on sales to minors (n = 1071)

	ED consumers (n = 202)	ED non-consumers (n = 869)	p-value (χ^2^)
**Age – years**	13.6 ± 1.0	12.9 ± 1.2	< 0.001^a^

**Sex, n (%)**			
Girls	94 (46.5)	454 (52.2)	0.144
Boys	108 (53.5)	415 (47.8)	

**Residency, n (%)**			
Urban	150 (74.3)	655 (75.4)	0.741
Rural	52 (25.7)	214 (24.6)	

Energy drink recognition* n (%)			
Yes	152 (75.2)	680 (78.3)	0.003
No	12 (5.9)	15 (1.7)	
Do not know/Unsure	38 (18.8)	174 (20.0)	

**Siblings, n (%)**			
None	43 (21.3)	127 (14.6)	0.035
Older siblings	77 (38.1)	329 (37.9)	
Younger siblings	57 (28.2)	321 (36.9)	
Both older and younger siblings	25 (12.4)	92 (10.6)	

a Mann Whitney U Test;

** Response to the following question “In your opinion, are energy drinks and isotonic drinks the same beverages?”

Among ED consumers, several statistically significant differences were observed between boys and girls (Table 3). Significantly more girls than boys had difficulty distinguishing between EDs and isotonic drinks (36.1% vs 14.8%). Both boys and girls preferred sugarfree EDs (58.9%) over those with sugar (41.1%), but boys selected sugar-free drinks significantly more often than girls (63.0% vs 54.3%). Slightly more than half of ED consumers (55.9%) reported that they never consume EDs before physical activity. Among those who consumed EDs before activity, the most common response was approximately once every four training sessions (37.1%), whereas the least common was ‘every other training session’ (6.9%). Girls reported consuming EDs in the context of physical activity significantly more often than boys (44.7% vs 30.6% for the answer ‘once every four workouts’ and 9.6% vs 4.6% for the answer ‘every other workout’).

**TABLE 3:** Characteristics of energy drinks (EDs) consumers studied in 2025, after implementation of the ban on sales to minors, by sex (boys and girls) (n = 202)

	Total (n = 202)	Boys (n = 108)	Girls (n = 94)	p-value (χ^2^)
Energy drink recognition* (%)				
Yes	75.2	85.2	63.8	0.002
No	5.9	4.6	7.4	
Do not know/Unsure	18.8	10.2	28.7	

**Do you usually chose ED (%)**				
With sugar	41.1	37.0	45.7	0.043
Sugar free	58.9	63.0	54.3	

**Frequency of ED use before exercise (%)**				
Never	55.9	64.8	45.7	0.021
Sometimes (once in 4 training session)	37.1	30.6	44.7	
Often (every other training) & Always	6.9	4.6	9.6	

* Response to the following question “In your opinion, are energy drinks and isotonic drinks the same beverages?”.

### Changes in Energy Drink Consumption and Usage Contexts Before and After the Sales Ban

**FIG. 2:**
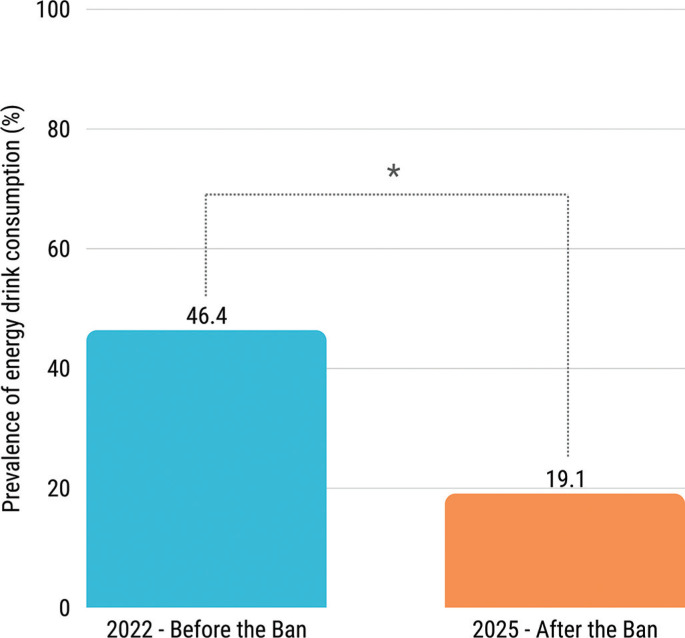
Prevalence of energy drink consumption among physically active adolescents before (blue) and after (orange) the introduction of the sales ban for minors. Statistically significant differences (p < 0.05, χ^2^ test) are indicated by *.

**FIG. 3:**
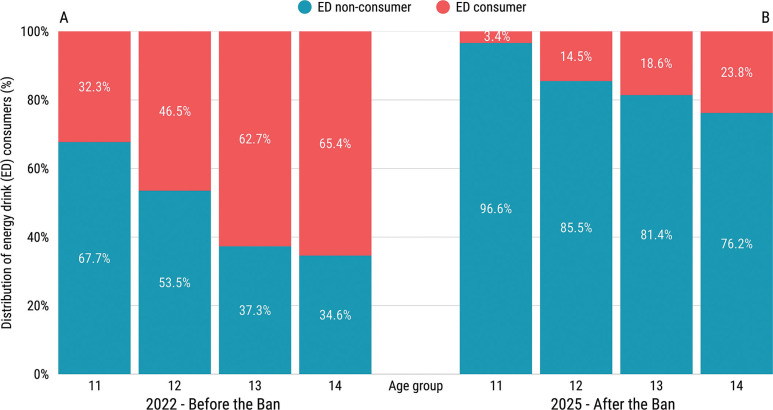
Distribution of energy drink (ED) consumers among various age groups – comparison between the time before (A) and after (B) the sales ban to minors. χ^2^ test revealed statistically significant differences in the proportion of ED consumers between 2022 and 2025 in all age groups (p-value < 0.001 for all comparisons).

The prevalence of ED consumption among physically active adolescents decreased significantly from 46.4% in 2022 to 19.1% in 2025 following the introduction of the sales ban for minors (p < 0.001; OR = 0.27, 95% CI: 0.23–0.33; Figure 2). Both before the ban (Figure 3A) and after the ban (Figure 3B), the percentage of ED consumers increased with age. Before the ban, the percentage of consumers ranged from 32.3% among the 11-year-old group to 65.4% among the 14-year-old group. After the introduction of the sales ban, the lowest percentage was also observed among the 11-year-old group (3.4%), while among the 14-year-old group it reached 23.8%. The χ^2^ test revealed statistically significant differences in the proportion of ED consumers between 2022 and 2025 in all age groups (p < 0.001). The post-ban study also included data on 15-year-olds, among whom the percentage of consumers was 38.1%.

**FIG. 4:**
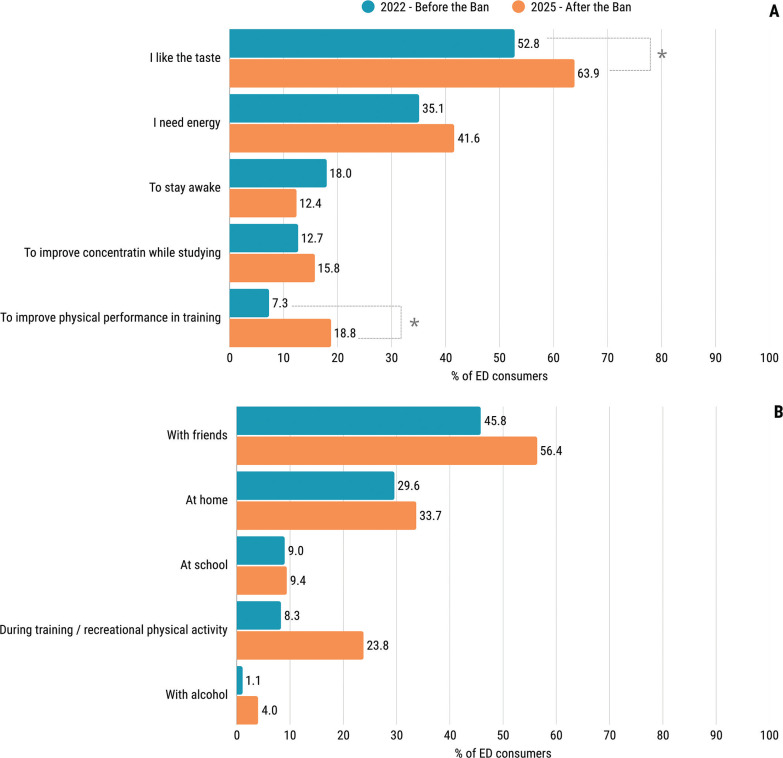
Comparison of reported reasons (A) and circumstances (B) of energy drink (ED) consumption among consumers before and after the sales ban to minors. Statistically significant differences (p < 0.05, χ^2^ test) are indicated by *.

The most frequently reported reason for ED consumption, both before and after the ban, was their taste (52.8% and 63.9%), as shown in Figure 4A. Significantly more consumers selected the response “I like the taste” after the sales ban than before (p = 0.007). In the 2025 study, a significantly higher proportion of consumers selected the response “to improve physical performance in training” compared with the 2022 study (p < 0.001). With regard to the circumstances of ED consumption (Figure 4B), both studies found that consumption with friends (45.8% before the ban, and 56.4% after the ban, p = 0.009) and at home (29.6% before the ban, and 33.7% after the ban, p = 0.305) were the most common. Significantly more participants reported consuming EDs during training in the 2025 study compared with the 2022 study (23.8% vs. 8.3%, p = 0.002). An increase was also observed in the combination of EDs and alcohol, from 1.1% before the ban to 4.0% after the ban (p = 0.016).

## DISCUSSION

To our knowledge, this is the first study to compare the prevalence, motives, and circumstances of ED consumption among adolescents before and after the introduction of a sales ban for minors. We demonstrated a significant reduction in ED consumption among physically active adolescents following the introduction of the sales ban, thereby confirming partial effectiveness of the implemented policy. What remains unchanged, regardless of the policy introduced, is that the frequency of ED consumption increases with age, as do the motives and circumstances surrounding it.

Our results are in line with those of a recently published study from Poland involving 999 high school students [15]. Even though 52.5% of study participants aged 15–17 years reported continuing to consume EDs after the ban, 68.9% of them declared a reduction in consumption as a result of the new policy. In a newly published study conducted after the introduction of the ban [16], the proportion of ED consumers among adolescents aged 12–17 years was 41.1%, exceeding the prevalence observed in our study. This difference may reflect the age composition of the samples: Wierzejska et al. [16] included older adolescents and, as in our analyses, reported that consumption increased with age. In that study, 97.3% of respondents were aware of the introduction of the ban. In a study of a representative sample of Polish adults (n = 1121) from 2024, it was demonstrated that 87.2% of respondents supported the sales ban to minors, however slightly less than a half (45.6%) considered it to be effective [17]. Moreover, 68.7% of respondents agreed that additional restrictions such as limiting advertising, are needed. Similar policy was introduced in Lithuania in 2014, Latvia in 2016 and Belarus in 2021. Yet the lack of research assessing its effectiveness prevents comparisons with our findings. However, it is worth emphasizing, that in countries where no legal measures have been taken to limit ED consumption among young people, there has been a steady increase in the consumption of these drinks [18, 19].

As shown in our research, the introduction of a sales ban did not alter the most commonly reported motives or contexts of ED consumption among physically active adolescents. Young people continued to select these beverages primarily for their taste and to consume them in the company of friends; in the post-ban survey, a significantly greater proportion of respondents endorsed these reasons than in the pre-ban survey. Previous research demonstrated that peer pressure is one of the key factor determining young people’s willingness to choose EDs [20]. Adolescents’ belief that EDs are ‘cool’ results from advertising and marketing, which is mainly targeted at this group. In particular, catchy advertising slogans and sponsorship of extreme sports events by popular brands make young people eager to consume EDs and to view them as desirable. A study involving young adults (aged 18–24) showed that just 8 minutes of viewing ED advertising materials increased positive feelings towards these drinks and increased the desire to purchase them [21]. It can be hypothesised that if this effect was observed in cognitively developed individuals, it may be even stronger in children and adolescents. Therefore, a reasonable next step to further reduce ED consumption among young people seems to be to restrict advertising of these products. Following the introduction of the ban, a significantly greater proportion of participants reported consuming EDs to enhance training performance compared with the pre-ban assessment; this shift likely reflects higher training loads in the post-ban cohort, yet remains concerning as it indicates a failure to distinguish EDs from isotonic sports beverages.

We also found that there were significantly more only children among ED consumers than in the non-consumer group. Our results in this respect are surprising because initially, we assumed that having siblings, especially older ones, would be a factor favouring EDs consumption, as consumption of these drinks increases with age. Other authors have shown for example that having siblings is associated with higher nutritional risk, such as greater consumption of processed foods and fast food [22–24]. This may be due, for example, to the reduced amount of attention that parents can devote to their children when they have many children. On the other hand, however, the large amount of attention that only children may receive from their parents can be associated with pressure to perform well academically [25, 26], and EDs may be used in response to this to cope with fatigue and stress. Young people without siblings may also seek acceptance outside the family home more often than children who have siblings [27], and ED consumption is perceived as appealing by their peers.

Several prior studies have indicated that boys consume EDs more frequently than girls [28–30]. Our earlier research involving physically active children corroborated this pattern [11]. In contrast, in the present study we found no statistically significant differences between girls and boys in the frequency of ED consumption. The increase in the proportion of girls in the EDs consumer group over the years has also been demonstrated in comparative study of Finnish teenagers between 2014 and 2018 [18], as well as in Polish study from 2022 on a representative sample of 5395 adolescents [31]. This shift may reflect changing norms and more similar leisure habits, so ED use – once more typical of boys – has become increasingly common among girls. Furthermore, following the introduction of the sales ban, we observed a significant increase in the proportion of consumers reporting ED use during training and for improved physical performance. Girls were significantly more likely than boys to report ED use in physical-activity contexts, a pattern that may be partly explained by more frequent confusion of isotonic beverages with EDs among girls. Problems with distinguishing the two drinks have already been reported by other researchers [32, 33]. This poses a significant risk: difficulty distinguishing isotonic from energy drinks can lead to unintentional, rapid consumption of high caffeine doses.

Adolescents in our sample more often selected sugar-free products than sugar-containing alternatives. This pattern has potential benefits – for example, reducing the risk of dental caries [34]. However, it also carries potential drawbacks: the physiological effects of non-nutritive sweeteners, particularly at higher cumulative intakes, remain uncertain in youth [35]. Given that many adolescents also consume other sweetener-containing products (e.g., high-protein foods and beverages), overall exposure may be substantial, with possible unfavourable effects on the microbiome [36]. Additionally, according to the 2023 WHO guideline, non-sugar sweeteners should not be used to control body weight in children [37].

Since bans on the sale of EDs to minors are a relatively new method of addressing ED consumption among adolescence, first introduced in Lithuania in 2014, there are no studies assessing their effectiveness [38]. The mere introduction of the ban probably helped raise awareness – particularly among parents with limited nutrition knowledge who previously did not recognize the risks associated with EDs. However it can be assumed that, in addition to the beneficial effects, they may have also a forbidden-fruit effect, thereby increasing the attractiveness and desirability of these drinks among teenagers, as with other bans, such as those on e-cigarettes [39]. In view of the above, it seems that interventions in the form of legal prohibitions should be accompanied by other actions, such as educational campaigns.

### Strengths and Limitations

The present study has several strengths, including a large number of participants (n = 1083), representation of all voivodeships, and the fact that it was conducted anonymously, which may have reduced social desirability bias. An additional strength is the methodological preparation: a pilot study was carried out in a smaller group to evaluate the questionnaire, and the survey itself was based on a validated tool used in the EFSA report [3]. Moreover, the study design was developed specifically to allow comparison before and after the introduction of the sales ban for minors. While other studies have already been conducted in Poland following the introduction of the sales ban for minors [15, 17], to our knowledge this is the first study worldwide to directly compare energy drink consumption before and after the implementation of such a policy. This provides unique evidence on its potential effectiveness.

Nevertheless, the results reported herein should be considered in the light of some limitations. The cross-sectional design precludes causal inference, and the post-ban sample comprised more physically active adolescents than the pre-ban sample – a group likely to have greater nutrition awareness – which could partly account for the differences observed. It should also be noted that the participants surveyed before and after the ban were not the same individuals, and the age ranges in the two studies were not fully identical (10–14 years in 2022 vs. 11–15 years in 2025), which limits the possibility of direct comparisons between the two datasets. In addition, methodological differences between the studies, including how ED consumers were defined, further limit the comparability of the findings; however, the adjusted definition was informed by insights from the pilot study. The sample was not nationally representative and included only adolescents engaged in sports training, so the results may not generalize to all Polish adolescents. Although the survey was anonymous, underreporting of ED consumption cannot be excluded; participants may have been reluctant to disclose prohibited behavior given awareness that sales to minors are banned in Poland.

## CONCLUSIONS

The significant decline in the proportion of EDs consumers suggests that the implemented policy might have been effective, albeit to a limited extent. However, the ban on the sale of EDs to minors did not alter age-related trends or the circumstances and motives for consumption. To achieve a greater impact on ED consumption behavior and reduce ED intake, educational campaigns for students, parents, and teachers should be implemented. Although the magnitude of the pre- versus post-ban difference observed here suggests that the policy may have contributed to reduced consumption, this study was conducted only among a physically active sample. Furthermore, the participants surveyed before and after the ban were not the same individuals, which limits the possibility of drawing direct conclusions, and it is also important to acknowledge the methodological differences in how consumers were defined. Therefore, this hypothesis requires confirmation in representative cohorts and through longitudinal evaluations including both physically active and inactive individuals.

## Supplementary Material


