# Supplementary material for: Before and after the ban: energy drink consumption among physically active Polish youth
Source: Biol Sport. 2025 Sep 9;42(4):381–92. doi: 10.5114/biolsport.2025.156871 (PMC12712672; doi:10.5114/biolsport.2025.156871)
Supplement: Supplementary file 1 [file JBS-42-4-57227-s1.pdf]

SUPPLEMENTARY MATERIAL

Supplementary File 1

Questionnaire on Energy Drink Consumption

(Students aged 11–15 years)

We are conducting research about adolescents' habits related to energy drink consumption and their attitudes toward these products. Your responses are anonymous and confidential and will be used for scientific purposes only.

Please select one or more answers as instructed for each item.

Please indicate your gender:

- ☐ Boy
- ☐ Girl
- ☐ Other / prefer not to say

Please provide your year of birth: ______________________________

Place of residence:

- ☐ City/town
- ☐ Village

Please select the province (voivodeship) where you live:

☐ Lower Silesian	☐ Subcarpathian
☐ Kuyavian-Pomeranian	☐ Podlaskie
☐ Lublin	☐ Pomeranian
☐ Lubusz	☐ Silesian
☐ Łódź	☐ Świętokrzyskie
☐ Lesser Poland	☐ Warmian-Masurian
☐ Masovian	☐ Greater Poland
☐ Opole	☐ West Pomeranian

Do you have siblings?

- ☐ No, I am an only child
- ☐ Yes, I have older sibling(s)
- ☐ Yes, I have younger sibling(s)
- ☐ Yes, I have both older and younger sibling(s)

Do you currently participate in organized sports activities (not including regular physical education classes)?

- ☐ Yes
- ☐ No

How many hours per week do you spend on physical activity? Include physical education classes, organized sports, cycling in your free time, etc. ______________________________

In your opinion, are isotonic drinks and energy drinks the same beverages?

- ☐ Yes
- ☐ No
- ☐ I don't know / hard to say

Information: Energy drinks are non-alcoholic, usually sweetened beverages that contain caffeine and other stimulants that reduce the feeling of fatigue.

In the past 6 months, have you consumed energy drinks?

- ☐ Yes
- ☐ No

Over the past 6 months, how often (on average) have you consumed energy drinks?

- ☐ Less than once a month
- ☐ 1–3 times per month
- ☐ 1–2 times per week
- ☐ 3–4 times per week
- ☐ 5–6 times per week
- ☐ Once a day or more often

Do you usually choose energy drinks that are:

- ☐ With sugar
- ☐ Sugar-free

Do you ever mix energy drinks with alcohol?

- ☐ Yes
- ☐ No

What are the main reasons you consume energy drinks? (You may select any number of answers)

- ☐ I like the tast
- ☐ I need energy
- ☐ To stay awake
- ☐ To improve concentration while studying
- ☐ To improve physical performance during training
- ☐ Other (please specify): ______________________________

In what situations do you most often consume energy drinks? (You may select any number of answers)

- ☐ With friends
- ☐ At home
- ☐ At school
- ☐ During training / recreational physical activity
- ☐ With alcohol
- ☐ Other (please specify): ______________________________

How often do you consume energy drinks before, after, or during training and/or recreational physical activity?

- ☐ Never
- ☐ Sometimes (about once every four training sessions)
- ☐ Often (about every other training session)
- ☐ Always

SUPPLEMENTARY FILE 2

TABLE S1. Study participants by province of residence

Voivodeship	n	%
Lower Silesian	167	15.4
Kuyavian-Pomeranian	43	4.0
Lublin	83	7.7
Lubusz	26	2.4
Łódź	162	15.0
Lesser Poland	24	2.2
Masovian	74	6.8
Opole	109	10.1
Subcarpathian	45	4.2
Podlaskie	50	4.6
Pomeranian	53	4.9
Silesian	42	3.9
Świętokrzyskie	34	3.1
Warmian-Masurian	91	8.4
Greater Poland	38	3.5
West Pomeranian	42	3.9
**Total**	**1083**	**100**

Articles published in the Biology of Sport are licensed under an open access Creative Commons CC BY 4.0 license.
